# The diagnostic potential of miR-196a-1 in colorectal cancer

**DOI:** 10.1186/s12885-024-11881-y

**Published:** 2024-02-01

**Authors:** Bayan Mehrjoei, Lida Haghnazari, Homayoon Bashiri, Nayebali Rezvani

**Affiliations:** 1 Department of Clinical Biochemistry, Kermanshah University of Medical Sciences, Kermanshah, Iran; 2 Imam Reza Hospital Kermanshah University of Medical Sciences, Kermanshah, Iran

**Keywords:** Colorectal cancer, Molecular diagnostics, miR-196a-1, *HOXD8* gene, Biomarkers

## Abstract

**Background:**

Colorectal cancer (CRC) is a common malignancy worldwide. MicroRNAs (miRNAs) are important epigenetic alterations that notably impact various physiological and pathological processes by acting as negative regulators of gene expression. Furthermore, they have a vital function in different types of cancers, including CRC. In this research, we evaluated, for the very first time, the expression levels of miR-196a-1 in the tissue and plasma of patients with CRC and also *homeobox D8* (*HOXD8*) as the target gene.

**Materials and methods:**

This study included a collection of 220 plasma and tissue samples from 55 patients diagnosed with CRC, as well as 55 healthy individuals matched by age and sex. Total RNA was extracted from plasma and tissue samples, and then polyadenylation and cDNA synthesis were performed. The expression levels of miR-196a-1 and *HOXD8* as target gene was evaluated by quantitative RT-PCR (qRT-PCR) assay. We compared the diagnostic value of plasma miR-196a-1 with that of the circulating tumor markers CA19-9 and CEA using a Receiver Operating Characteristics (ROC) analysis. The association of miR-196a-1 with clinicopathological characteristics was assessed in tissue and plasma samples from patients with CRC.

**Results:**

Our data demonstrated that the expression levels of miR-196a-1 in the tissue and plasma samples of CRC patients were 11.426- and 11.655-fold higher, respectively than those in adjacent normal tissue and plasma samples from normal subjects (*p* < 0.001). Through ROC curve analysis, it was identified that the sensitivity and specificity of miR-196a-1 for tissue samples, with an AUC of 0.925, were 89% and 98%, respectively. In addition, the sensitivity and specificity for plasma samples with an AUC of 0.801 were 70% and 98%, respectively. These findings reveal that miR-196a-1 is a useful biomarker for discriminating cases from controls. Furthermore, the expression of *HOXD8* was not significantly altered in tumor tissue samples compared to adjacent normal tissues (*P* > 0.05).

**Conclusions:**

These results show that miR-196a-1 has an oncogenic impact and plays a significant role in CRC development. The results also indicate that miR-196a-1 could serve as a novel noninvasive biomarker for the detection of CRC.

## Background

Among all types of malignant neoplasms, colorectal cancer (CRC) is presently the third most prevalent, while it ranks second among the leading causes of cancer-related fatalities worldwide [1]. Annually, more than one million individuals are diagnosed with this disease [2]. In Iran, CRC is the second most common cancer in women after breast cancer and the fourth most common cancer after stomach, bladder, and prostate cancers in men [3]. Unfortunately, During the last 25 years, there has been a noticeable rise in CRC prevalence, especially among younger age groups [4]. Despite the substantially improved five-year overall survival rate and major advances in diagnostic and therapeutic developments for CRC patients in recent decades, CRC remains a prominent cause of cancer-related death and a serious public health concern [5, 6]. Therefore, it is important to understand the pathological and molecular mechanisms that impact the tumorigenesis and progression of colorectal cancer, as well as to identify biomarkers for the prevention and early detection of this disease [7, 8]. MiRNA molecules modulate gene expression post-transcriptionally [9]. MiRNAs exhibit both tumor suppression and oncogenic activities by binding to the 3′-UTR of their target mRNAs, which can result in translational repression or degradation of mRNA in several cancer types [10].

MiRNAs are involved in various biological processes, including cell proliferation, differentiation, apoptosis, and development [11]. Aberrant expression of different microRNAs has been observed in the serum, plasma, and tissues of CRC patients. It has been proposed that miRNAs could have significant involvement in CRC [12]. The evolutionary conservation of the miR-196 gene family remains intact and consists of three members, namely, miR-196a-1, miR-196a-2, and miR-196b. These miRNAs are situated inside *homeobox (HOX)* gene clusters. The mature nucleotide sequences of miR-196a-1 and 2 are identical, whereas they have only one nucleotide difference from mature miR-196b. In humans, the location of the miR-196a-1 gene (Accession Number MI0000238) is situated in a region between *HOXB9* and *HOXB10* on Chr. 17q21.32. Previous investigations have revealed that members of the miR-196 family play vital roles in the pathogenesis and development of cancer. They have been aberrantly expressed in various cancers, such as melanoma, gastric, pancreatic, lung, glioblastoma, leukemia, and colorectal cancer [13–16]. *HOX* genes have various roles in the early development of embryos and organs. They are frequently linked to the development and progression of tumors [17–19]. *HOXD8* is a member of the *HOX* gene family and is located on chromosome 2q. These genes are commonly deregulated in various malignancies, including lung cancer, hepatocellular carcinoma, neuroblastoma, pediatric brain tumors, glioma, and colorectal cancer. *HOXD8* is implicated in numerous biological processes, such as proliferation, cell differentiation, cell apoptosis, cell cycle regulation, regulation of tissue migration, and organ morphogenesis during embryogenesis. *HOXD8* is acknowledged as a downstream target of many miRNAs, including miR-196a [20–22].

Previous studies have highlighted that miR-196a-1 can serve as a biomarker with oncogenic potential for several diseases, including leukemia, gastric cancer, and glioma. The expression of *HOX* genes in leukemia has been correlated with miR-196a-1 [23–25]. Also, *HOXD8* has been identified as a downstream target of miR-196a, and it was found that the expression level of *HOXD8* was downregulated in colorectal cancer tissues. Therefore, this indicates that miR-196a has a potential oncogenic function in this disease [26]. As miR-196a-1 belongs to the miR-196 gene family, and according to the fact that no study has been conducted on the function of miR-196a-1 in colorectal cancer, the purpose of the current investigation was to determine the expression levels of miR-196a-1 and its target gene, *HOXD8*, in tissues and plasma samples of patients with CRC. Additionally, a comparison of the diagnostic sensitivity and specificity of plasma miR-196a-1 with CEA and CA19-9, which are common CRC biomarkers, was performed. Furthermore, the connection between the expression of miR-196a-1 and the clinicopathological characteristics of CRC patients was thoroughly examined.

## Materials and methods

### Patients and clinical specimens

A total of 55 pairs of tumor tissue samples matched with adjacent normal tissue, as well as 55 plasma samples from patients with CRC (25 males and 30 females) with a mean age of 57.92 ± 17 and 55 plasma samples from normal subjects (27 males and 28 females) with a mean age of 57.94 ± 12, were obtained from patients and normal subjects who were admitted to Imam Reza Hospital in Kermanshah, Iran. Immediate preparation and storage at -80 °C were performed for all samples. None of the participants received chemotherapy, radiotherapy, or adjuvant treatment before surgery.

Criteria such as confirmation of a person’s disease by the results of colonoscopy and pathology tests, not having a history of chemotherapy, radiotherapy, adjuvant treatment, and other malignant neoplasms before surgery and also patients who agreed to participate in the study were the entry criteria for the patient group. Also, Patients were excluded from the study if they had received preoperative chemotherapy or radiation therapy or had a previous history of malignant tumors. On the other hand, among the inclusion criteria for the control group, we can mention the normality of the colonoscopy report, no history of malignancy, inflammatory bowel disease, ulcerative colitis, Crohn’s disease, and lack of chemotherapy and radiotherapy. Moreover, the adjacent tissue samples were excluded if they exhibited any abnormalities or pathological features.

### Human plasma samples

Human blood samples were collected from patients with CRC and normal subjects and preserved in EDTA-K2 tubes. The samples were then centrifuged, and the plasma was isolated. All plasma samples were fresh frozen in two separate tubes using liquid nitrogen. They were then stored at a temperature of -80 °C until needed.

### Human colorectal cancer tissue samples

Samples of both tumor and adjacent normal tissues were obtained from patients with CRC during surgery. Adjacent normal tissue was obtained from a location at least 10 cm away from the cancer region. Liquid nitrogen was used to freeze the samples, which were then stored at -80 °C.

### RNA extraction and cDNA synthesis

Total RNA was extracted from plasma and tissue samples using the NucleoSpin® miRNA Plasma MN (Macherey-Nagel) RNA extraction kit and the favorgen mRNA kit (Cat. No: FAMIK002) following the manufacturer’s instructions, respectively. The concentrations and purity of total RNA in all samples were measured using a Nanodrop 1000 Spectrophotometer. Subsequently, polyadenylation and immediate cDNA synthesis were executed by a BON miR Kit (Bon Yakhteh, Tehran, Iran).

### Quantitative real-time PCR

Bonyakhte Company Tehran designed and synthesized primers specifically for genes, with *HPLC* grade for the amplification of the transcripts of the miR-196a-1 and *U6* genes as an internal control. Real-time PCR was conducted using an Applied Biosystems (ABI) 7500 Real-time system (Forster City, CA, USA) using the BON miR SYBR® green protocol (Bon Yakhteh, Tehran, Iran, BON209002). The qRT-PCR reaction mixture was 10 μl, containing ROX Reference Dye II (0.25 μl), Forward primer (0.5 μl), reverse primer (0.5 μl), cDNA (1 μl), PCR Master Mix (5 μl), and distilled H_2_O (2.75 μl). Amplification Reactions for microRNA were incubated in a 96-well plate at 95°C for 2 min for initial denaturation, followed by 45 cycles of 95°C for 5 s and 62°C for 20 s. The Primer sequences used in this study were as follows: *HOXD8*-forward: 5’**-**AGAAGAATCGAGGTTTCCCACG-3’,

*HOXD8*-Reverse:5’TCCTTTTTCGTTTCCCCGTCC-3’, *U6*-forward:

5’**-**CTCGCTTCGGCAGCACATA-3’, *U6*-Reverse: 5’**-**CGAATTTGCGTGTCATCCT-3’. The expression of miRNA-196a-1 and *HOXD8* was determined relative to *U6* using the 2^−ΔΔCt^ method.

### Common biomarker detection

The concentrations of CEA and CA19-9 in the plasma samples of patients with CRC and normal subjects were detected according to the instructions of the Liaison CEA kit (Cat. No: 314,311, Diasorin) using the Liaison® chemiluminescence analyzer (Diasorin, Saluggia, Italy).

### Statistical analysis

The results of real-time PCR were analyzed using real-time PCR software ABI 7500 SDS v1.3.1. Relative Expression Software Tool (REST2009) was employed to assess the relative expression of miR-196a-1 between patients with CRC and normal subjects. The sensitivity and specificity of miR-196a-1 expression levels in tissues and plasma as a diagnostic test, as well as the CEA and CA19-9 tests, were assessed using the ROC curve. All statistical tests were executed using SPSS v.16.0, and *P* values < 0.05 were considered statistically significant.

## Results

### Upregulation of miR-196a-1 expression in CRC tissue samples

**Fig. 1:**
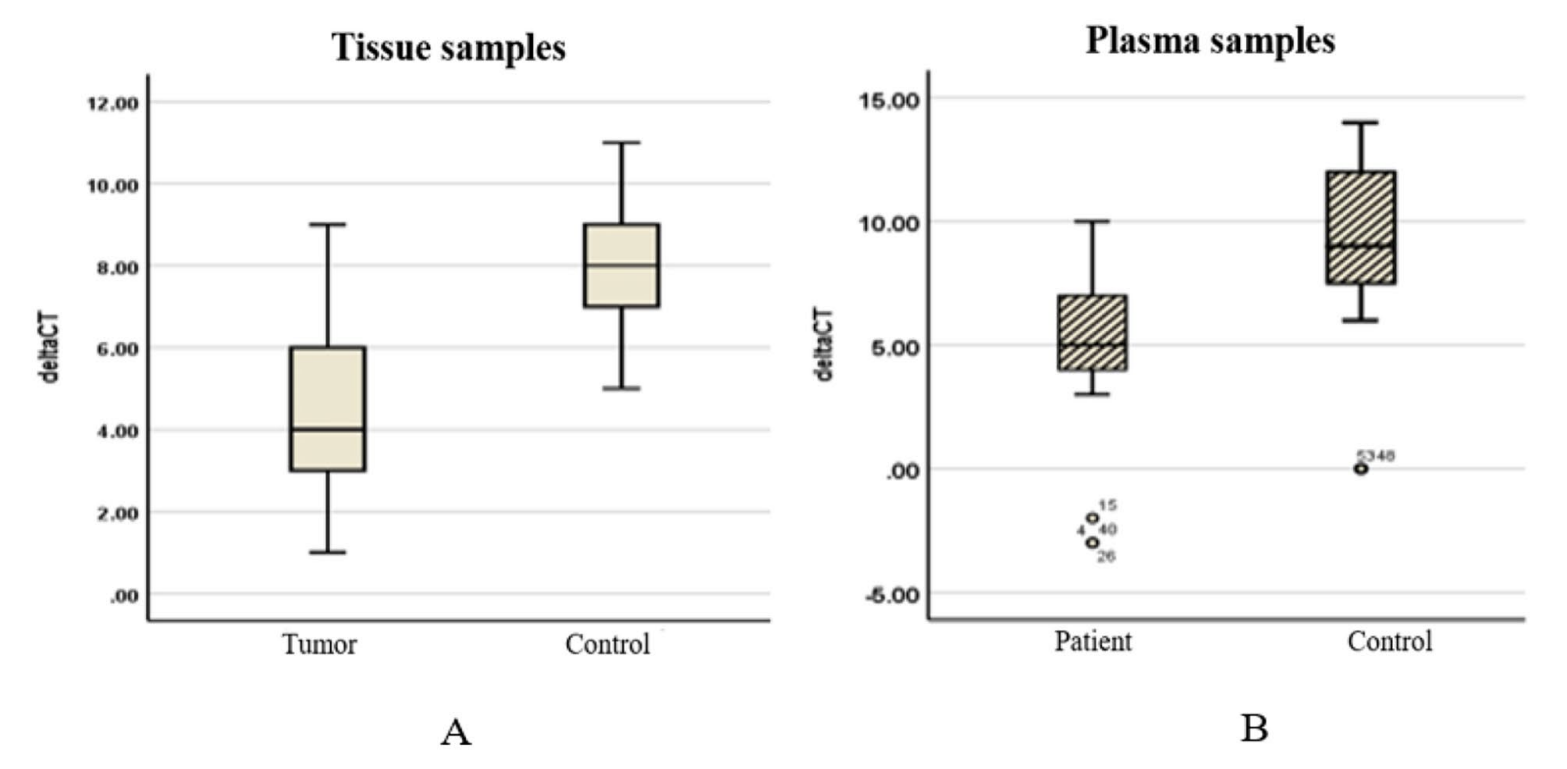
Box plot of the expression level of miR-196a-1 in the tissue and plasma of patients with CRC. (**A**) The expression level of miR-196a-1 was significantly increased in CRC tissues compared to adjacent normal tissues (*P* < 0.001). (**B**) The expression level of miR-196a-1 was significantly increased in the plasma of patients with CRC compared to normal subjects (*P* < 0.001)

In this study, miR-196a-1 expression levels were evaluated by qRT-PCR in 55 paired tumor tissue samples and adjacent normal tissues. Our study demonstrated that the miR-196a-1 expression levels were significantly increased by 11.426-fold in the tissue samples of patients compared to adjacent normal tissues (*P* < 0.001, CI: 0.016–1.024, Fig. 1A).

### Upregulation of miR-196a-1 expression in CRC plasma samples

The findings revealed that the expression level of miR-196a-1 was significantly increased by 11.655-fold in 55 plasma samples from patients with CRC compared to 55 plasma samples from normal subjects (*P* < 0.0001, CI: 0.016–2.048).

### Expression of miR-196a-1 and clinicopathological characteristics

The relationship between the expression of miR-196a-1 and clinicopathological characteristics in tissue and plasma samples of CRC patients was investigated and is summarized in Table 1 However, no statistically significant associations were observed between the expression of miR-196a-1 and age, sex, clinical stage, tumor location, tumor size, or tumor type (*P* > 0.05).

**Table 1:** The relationship between miR-196a-1 expression and clinicopathological characteristics in tissue and plasma samples from CRC patients

Variable	The expression of miR-196a-1 in tissue			The expression of miR-196a-1 in plasma	
	Number	Mean ± SD*	*P* value	Mean ± SD	*P* value
**Age(years)**					
≤50	15	5.09 ± 1.69	0.872	4.98 ± 2.85	0.912
>50	40	5.18 ± 1.78		4.68 ± 2.89	
**Sex**					
Male	25	4.88 ± 1.91	0.305	4.31 ± 3.18	0.905
Female	30	5.18 ± 1.74		4.56 ± 2.98	
**Tumor type**					
Adenocarcinoma	32	4.48 ± 1.84	0.271	4.73 ± 2.85	0.757
Mucinous	23	5.28 ± 2.28		5.07 ± 3.83	
**Clinical stages**					
Stage I, II	37	4.52 ± 1.56	0.419	4.68 ± 3.41	0.41
Stage III, IV	18	5.05 ± 2.03		5.85 ± 1.95	
**Tumor size**					
≤5 cm	39	4.54 ± 1.97	0.862	4.47 ± 3.68	0.491
>5 cm	16	5.06 ± 2.023		5.21 ± 2.78	
**Tumor location**					
Distal	19	4.51 ± 2.46	0.663	4.88 ± 3.58	0.965
Proximal	36	4.81 ± 1.87		4.83 ± 3.18	

*Standard deviation

### The ability of miR-196a-1 expression level as a biomarker

**Fig. 2:**
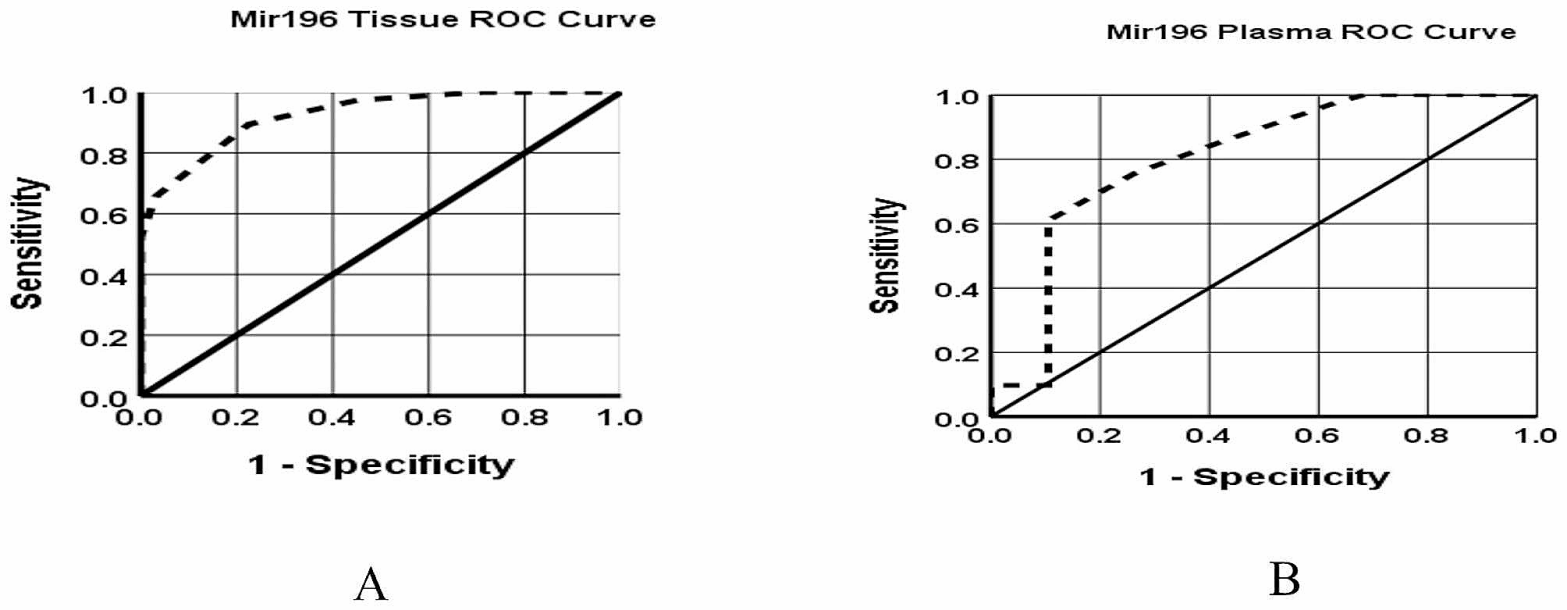
ROC curves of (**A**) miR-196a-1 expression in tissue, (**B**) miR-196a-1 expression in plasma

In addition, we exploited the ROC curve to evaluate the ability of miR-196a-1 expression level in tissue and plasma samples to discriminate between CRC patients and controls. The examination of the ROC curve showed that the sensitivity and specificity of miR-196a-1 for tissue samples with an AUC of 0.925 (95% CI: *P* <0 0.001) were 89% and 98%, respectively. Similarly, the sensitivity and specificity for plasma samples with an AUC of 0.801 (95% CI: *P* <0 0.001) were 70% and 98%, respectively (Fig. 2). According to the results, miR-196a-1 could be used as a useful biomarker to discriminate patients with CRC from controls.

### ***HOXD8*** gene expression as the target gene of miR-196a-1

Using real-time PCR, we measured the expression of the *HOXD8* gene in tissue samples of patients with CRC and compared it to adjacent normal tissues. According to the results, there was no significant difference in *HOXD8* expression between tumor tissue samples and adjacent normal tissues (*P* > 0.05).

### Evaluation of CEA, CA19-9 and miR-196a-1 expression levels

Using the ROC curve, we tested miR-196a-1 expression in plasma samples as a diagnostic biomarker for CRC patients in comparison to the common CRC biomarkers CEA and CA19-9. The ROC curve showed that the sensitivities of CEA, CA19-9, and miR-196a-1 were 46.8%, 43.2%, and 70%, respectively, while the specificities of CEA, CA19-9, and miR-196a-1 were 100, 56.8% and 98%, respectively. The results revealed that miR196a-1 could be used as a useful diagnostic biomarker for the detection of CRC patients.

## Discussion

MiRNAs are remarkably stable and even in RNase-rich environments protected from RNase degradation, which may be because of their small size. One can easily extract miRNAs from formalin-fixed paraffin-embedded (FFPE) tissue specimens and biological fluids such as blood, urine, and feces. These molecules are secreted by cancer cells in the circulatory system and gastrointestinal tract. MiRNA levels are independent of age and sex [27, 28]. Recent research has proven that miRNAs can function as potential noninvasive screening biomarkers for the detection of patients with colorectal cancer. Elevated levels of miR-196a-1 have been observed in both glioma and gastric cancer, as reported in earlier studies. Additionally, this miRNA has been found to have oncogenic potential in leukemia and has shown a clear association with the expression of *HOX* genes [23–25]. Nevertheless, the function of miR-196a-1 in colorectal cancer has yet to be determined. Therefore, the purpose of this research was to assay the involvement of miR-196a-1 in colorectal cancer.

The results of our current research revealed that the expression of miR-196a-1 was significantly upregulated in tissue and plasma samples from patients compared with controls. Consistent with our results, Feng et al. [24] recently reported that the expression of exosomal miR-196a-1 displayed a substantial increase in gastric cancer (GC). Furthermore, this increased expression was correlated with poor survival in this disease. Additionally, miR 196a-1 was responsible for driving the invasion and metastasis of GC cells specifically toward the liver by downregulating *SFRP1.* Our data indicated that miR-196a-1 had higher sensitivity and specificity than CEA and CA19-9 in plasma samples. The outcomes of the present research revealed that *HOXD8* was not altered in CRC tissues.

Several studies have demonstrated that the *HOX* gene is aberrantly expressed in numerous cancers and plays an important role in tumor suppression. For example, Mansour et al. [21] showed that *HOXD8* expression in colorectal cancer tissues is downregulated. Furthermore, the expression of this gene significantly inhibits cell proliferation, colony-forming ability, and invasion while inducing cell apoptosis in CRC cells. In another study, Sun et al. [22] demonstrated that *HOXD8* is downregulated in hepatocellular carcinoma. Additionally, WEN et al. [29] reported that *HOXD8* is significantly downregulated in breast cancer, leading to the inhibition of the cell cycle, migration, and invasion. Yao et al. [30] discovered a significant downregulation of the *HOX* gene in gastric cancer. In a recent study, Liu et al. [31] obtained similar results to the above studies in colorectal cancer. Furthermore, studies have demonstrated that miR-196a plays a role in regulating CRC metastasis by targeting *HOXD8* [26].

In the current study, we investigated the expression of *HOXD8* in tissue and plasma samples of CRC patients, and our findings showed no significant difference in *HOXD8* expression. These results are inconsistent with the findings of the previously reviewed studies. However, the KANAI study was consistent with our study and showed that *HOXD8* gene expression may vary in different parts of the intestine. Specifically, in tumor tissues of the left-side large intestine, the level of *HOXD8* gene expression was lower than in normal mucosa of the same side, while there was no significant difference in the expression of this gene in tumor tissues of the right-side large intestine compared to its normal mucosa tissues. This could be attributed to the fact that cancers originating from the right side of the large intestine exhibit distinct genetic and epigenetic statuses compared to those arising from the left side of the large intestine [32]. The results of gene expression studies in colorectal cancer can vary among different studies due to sample and biological variability. Indeed, the composition of study samples, such as the stage of cancer, tumor heterogeneity, patient characteristics, and treatment history, can lead to differences in observed gene expression patterns. Statistically, it was also possible that if the number of samples was greater, it would affect the significance of the variables [33]. Additionally, gene expression can be influenced by factors such as the tumor microenvironment and genetic and epigenetic modifications.

## Conclusions

In summary, as the first study to assess the expression of miR-196a-1 in tissue and plasma samples of patients with CRC, our research discovered that the expression of miR-196a-1 in tissue and plasma was significantly upregulated in patients with CRC compared with normal subjects. These findings suggest that miR‑196a-1 can act as an oncogene in the pathological processes of CRC. Furthermore, the level of miR‑196a-1 was not connected with clinicopathological characteristics in CRC patients.

Furthermore, our data suggest that miR-196a-1 has the potential to serve as a noninvasive screening and diagnostic tool for the detection of CRC in comparison to common biomarkers in CRC, such as CEA and CA19-9, in both tissue and plasma.

### Limitations

A limitation of our study was the relatively small sample size. To strengthen the reliability and applicability of our findings, it is imperative to conduct further research with a larger sample size.

## Data Availability

All data generated or analyzed during this study are included in this published article.

## Abbreviations

CEA: Carcinoembryonic antigen
CRC: Colorectal cancer
*HOXD8*: *Homebox D8*
Mir-196a-1: MicroRNA196a-1
ROC: Receiver operating characteristic
RT‒PCR: Real-time polymerase chain reaction
